# On the Diseases and Derangements of the Nervous System

**Published:** 1841-07

**Authors:** 


### Art. XVI.

*On the Diseases and Derangements of the Nervous System.* By Marshall Hall, m.d. f.r.s.—*London*, 1841. 8vo, pp. 380.

There are several reasons why we do not enter upon a full examination of this work at present. In the first place, our available space would not allow us to treat the subject of it as fully as we could desire. Secondly, we have already in the present Number devoted an article of considerable length to nervous diseases. Thirdly, the author announces a much larger and more complete work on the subject, which, if not too long delayed, we would prefer waiting for, more particularly as the present volume, although containing some new points, is, in a considerable degree, made up of materials already before the profession in various publications by the author, the greater number of which have been noticed in this Journal. As, however, Dr. Hall has done us the honour to give to our criticisms of his former works a very prominent place in the one before us, devoting the greater part of a somewhat long preface to reclamations from our judgments and opinions, in which he vents most bitter accusations of ignorance, prejudice, wilful misrepresentations, and all sorts of unworthy motives against the supposed authors of our reviews, we must here spare a few pages to the consideration of the chief points at issue between him and ourselves. Dr. Hall ought to receive this as a compliment at our hands, as he must be aware that we hardly ever take notice of the remonstrances of authors against our judicial awards, when it happens to us, as it must occasionally happen to every independent journalist, to form an estimate of their writings, different from that which they themselves entertain.

I. In our Third Volume pp. 29-39, Dr. Hall's Lectures on the Nervous System and its Diseases were reviewed. It was there pointed out that the class of actions to which he had given the name of *excito-motor* corresponded with that denominated *sympathetic* by Whytt and other physiologists; but it was freely admitted that merit was due to him for "fixing the attention of physiologists upon them, and on the agency of the spinal cord in producing them; and likewise in casting doubts on the doctrine of their dependence on sensation." On this head we should have expressed ourselves more strongly had not Dr. Hall claimed too much, by asserting that the phenomena to which he was directing attention were "quite unknown to physiologists." The question whether or not these actions necessarily involve sensation was left *sub judice;* "whether they really indicate sensation or not," we observed, "can only be decided by observations in cases of injury or disease of the human body," and two cases were referred to in support of the negative. We *never denied* this part of Dr. Hall's doctrines, as he insinuates that we once did (p. xiii.); but it would have been most unphilosophical to have received them as established facts, upon the very insufficient evidence at that time adduced by him. Dr. Hall complained of our award; and we admitted into our next Number (vol. III. p. 577, et seq.,) his own statement of his claims; and what he intended to be a correction of our mistakes regarding them. To these his reviewer appended some remarks, which clearly showed that he had not in the least misapprehended the degree in which that portion of Dr .Hall's doctrines that might then be regarded as admissible, had been previously taught by Physiologists.

II. Dr. Hall's second Memoir was reviewed by us in our Number for April, 1838. We there entered fully into the scientific and historical merits of the question with no other view, we can most conscientiously affirm, than the elucidation of truth. The additional evidence adduced in the interval by Dr. Hall and others, and especially the publication of cases of the class just referred to, induced us at that time to express our full concurrence in the general fact asserted by Dr. Hall, that the class of actions termed *reflex* is dependent upon the spinal cord and its nerves alone, and *does not* involve sensation as a necessary element of its phenomena. We fully enquired into the history of this opinion, and showed that it had been nearly approached in the doctrines propounded by former physiologists; and we concluded with expressing our belief that Dr. Hall's "great merit consists in the harmonious combination of these doctrines into an uniform system, and the explanation of many phenomena which were not formerly regarded as explicable on such principles." (vol. V. p. 537.) To this opinion, in regard to the physiological principles respecting which it was then expressed, we still most firmly adhere; but, as we were not at that time disposed to consider as proved the anatomical distinctness of the excito-motor and sensori-volitional systems of nerves, we did not attribute to Dr. Marshall Hall any merit as the discoverer of it. We did not by any means assert, however, that this last doctrine was false, but left the question open to be decided by future evidence. It is to this article that Dr. Hall's most frequent reference is made, as one calculated to do him serious injury in the eyes of the scientific world. Yet he has nowhere entered upon that calm examination of its asserted fallacies, which alone can justly entitle him to assail it with the violence he has employed. We will venture to say that, by the arguments there adduced in favour of a part of his views, the minds of the profession were better prepared to receive the whole at a subsequent period than they would have been if we had not noticed the subject at all; so that the critique was, after all, of service to him rather than otherwise.

At the conclusion of the same Number which contained this review, we inserted the chapter from Prochaska on the Reflex Function (which had only just become known to us,) with the heading "Complete Anticipation of Dr. M. Hall's Doctrine of the Reflex Function." The essential correctness of this statement, we are still prepared to maintain; and we have only to remind our readers, that it refers, *not* to the distinct excito-motor system of nerves, of whose existence we were not at that time convinced,—but to the Reflex Function alone, of which Prochaska had given an account that proved that he well understood it.

III. When additional evidence was adduced in behalf of Dr. Hall's doctrine of the structural distinctness of the excito-motor system, we took the earliest opportunity of recording our adhesion to it, and of making the *amende honorable* to Dr. Hall, which we did in the following words: "As to Dr. Hall's *entire originality* in this part of his doctrines we never expressed the slightest doubt; and we now are quite willing to accord to him the credit of having made a most important advance in nervous physiology, not only in the manner already stated, but by the discovery of a previously unsuspected fact, which, when placed entirely beyond doubt, will take the rank of those established by Sir C. Bell." (vol. VIII. p. 511.)

IV. In a subsequent article (vol. IX. p. 96, &c.) we contrasted Dr. Hall's discovery at greater length with those of Sir C. Bell, and the result appears to have been satisfactory even to him. (Preface, p. xi.) Yet he still pressed on us his own statement of his claims, to which, as before, we gave free admission. (vol. IX. p. 580.)

V. Our last notice (April, 1841,) refers principally to the *vis nervosa*, a question on which we have the misfortune to be still at issue with him.

Dr. Hall maintains that the physiological facts brought to light by him were sufficiently conclusive in respect to the structural distinctness of the system of excito-motor nerves from the sensori-volitional, to render additional evidence unnecessary; and seems to wonder that any physiologist could feel the necessity of *anatomical* confirmation to his views. To this we simply reply that, if such were the case, the great discoveries of Sir C. Bell are comparatively valueless; since they only confirmed—what had often been previously asserted on physiological grounds to the full as satisfactory as those on which Dr. Hall has proceeded—the distinctness of the sensory and motor nerves.

If our readers will refer to the second of the articles just enumerated, they will find (vol. V. p. 438,) that we there stated it as the published opinion of several physiologists, who admitted Dr. Hall's general doctrines on the physiology of reflex actions, that these *did not in themselves* warrant the recognition of a system of nerves *anatomically* distinct; and that the question might be considered an open one, to be decided by further evidence. We have reason to know that, in this view of the *then* state of the case, we expressed not only our own opinion and those of the authors cited, but of several other distinguished physiologists, including two whose opinion in favour of the general value of his discoveries Dr. Hall records (p. 232, note,) with great satisfaction, and whose opinion in *our* behalf we expect that he will hold in equal regard. Dr. Holland introduces the last chapter of his Medical Notes and Reflections, "On the present state of enquiry into the nervous system," with the following passage: "Those who desire to learn the actual state of knowledge in this complicated enquiry, will do well to refer to a series of most able articles in the British and Foreign Medical Review, embracing its relations to all other branches of physiology as well as to the higher and more general principles of inductive science." As the first edition of the work was published between the times of appearance of the Review of April, 1838, and that of January, 1840, it cannot be doubted that Dr. Holland meant to refer to the former and to the preceding articles. Dr. Sharpey also in his public lectures on physiology at University College, delivered in the spring of 1838, expressed opinions exactly conformable to ours.

Dr. Hall more than once taunts us with having changed our opinions as to the truth of his doctrines. Surely such a taunt comes with an ill grace from one whose great source of complaint against others is that they will *not* change theirs; and who has been so continually modifying his own during the last few years, that it would be very easy for us to point out apparent inconsistencies, much more numerous than those for which he attempts to bring us into odium. For example, between the publication of his first and of his second memoirs, he changed his opinion on the subject of the source of the respiratory movements; having in the former considered the medulla oblongata as their primum mobile; and in the latter announced as a new discovery that which was essentially the opinion of Whytt, that they are occasioned by a stimulus conveyed to the nervous centres, chiefly by the par vagum. And, in the volume now before us, he admits that the contractions of the œsophagus are *partly* reflex, (p. 71,) although he formerly maintained that they were independent of the excito-motor system. Now *we* do not consider it a fault in Dr. Hall that he has been induced by new evidence, or by a reconsideration of the old, to modify his first opinions; and we only ask for ourselves, and for other physiologists, the same liberty. And if he finds that they are willing to admit, upon evidence adduced by others, doctrines to which they refused assent when propounded by himself, the more dignified course would certainly be to express gratification in their conversion to the true faith, rather than to upbraid them with their previous incredulity. It cannot be too strongly impressed upon those who are eager in the propagation of what they believe to be novel and important truths, that the same facts will afford to different minds very different degrees of evidence of them. Wherever there is room for diversity of opinion, the variable constitution of the human mind is such, that diversities *will* be entertained; and we see no more hope of perfect conformity to a general standard in physiology than in religion. We are quite willing to admit that the array of numbers against any particular novel doctrine is no evidence of its falsity; for scarcely was an important truth ever announced that did not at first meet with general opposition. But when the subject is taken up by a number of independent enquirers, experienced in the scientific pursuit of truth, and possessing competent knowledge of its details, their general agreement in one decision does seem to us to indicate, either a fallacy in the original proposition, or a want of evidence sufficiently convincing to those who are acquainted with the general bearings of the question. That one or two individuals of previously unknown character might, from the first, give their assent to the new doctrines, can scarcely be regarded as any testimony in favour of them, or as any merit on their own part; since such assent might easily be obtained from those not versed in the question, by a one-sided statement of it. For ourselves we can only say that, looking back at what we have written on this *quæstio vexata*, there is not a position we have taken which we are not prepared to justify, as the most conformable to the *then* state of physiological knowledge.

We shall now briefly notice the more particular charges against us, contained in Dr. Hall's preface, and scattered through the volume. In regard to the history of the subject, several of the statements which we formerly made are so distorted that it would be difficult to recognize them again; and we shall content ourselves with requesting such of our readers as feel an interest in the discussion to refer to vol. V., pp. 532-57, for our opinion of the degree in which Dr. Hall had been anticipated by Flourens, and to vol. IX., p. 113, for a similar analysis in regard to Prochaska, and to decide for themselves whether we have made the slightest attempt to show that either of these physiologists had made any approach towards the discovery of a *distinct system of excito-motor* nerves, of which Dr. Hall charges us (at least we know of no other writer to whom he can refer, § 253), with attempting to transfer the merit to others. In order to prove our ignorance and malice, he has obtained from M. Flourens the following disavowal: "Votre beau système des nerfs *excitateurs*, *incidens*, et *réfléchis*, vous appartient bien, et comme grand fait spécial et déterminé, et comme vue d'un grand et nouvel ensemble de phénomènes." But in this he is fighting with a shadow entirely of his own evoking; for, from the moment when we were convinced that the doctrine was valid in itself, we have not hesitated to give the full credit of it to Dr. Hall; and not a word can he find in our previous criticisms showing the least tendency to deprive him of its paternity. As to the question how far M. Flourens had anticipated Dr. Hall in the more general doctrines of reflex action, our statements have never been impugned in any other way than by clamorous vituperation. We have given an abstract of what M. Flourens *does* say, and Dr. Hall replies by assertions as to what he *does not* say; and leaves it to be inferred that, because M. Flourens does not express his opinions in Dr. Hall's language, there was nothing common between the two. Surely the fairer way would be to take our assertions, sentence by sentence, if they are incorrect, and to prove their falsity from M. Flourens' own work. The opinions which we subjoined, as to the probable modification of M. Flourens' doctrines by Sir C. Bell's discoveries, we offered merely as an *opinion;* and there is not a word in M. Flourens' disavowal of the "distinct system," which in the least contradicts it.

The "vis nervosa" question, however, seems to be, in Dr. Hall's mind, the sorest point of the whole litigation between us. We really feel loath to inflict upon our readers any further discussion upon what may seem to be so personal a dispute. But we have found from Dr. Hall's last arguments, what we had all along suspected, that he does not understand the words in the ordinary sense; that, when he is talking of the *vis nervosa* of Haller, he really means something quite different. Referring to our former statement, that if the "*vis nervosa* of Haller be intended, this is alike the means by which volition, emotion, and simple reflex action, produce muscular movement." Dr. Hall remarks, "How does my young critic know this? it may be, or may not be so;" and he frequently elsewhere speaks of volition, emotion, and the vis nervosa, as the three modes by which the nervous system operates upon muscle. Now, *our* assertion is founded upon the simple fact, that Haller's own definition of the term does not admit its employment in any other sense than the one we have assigned to it. No reader of Haller's works can be ignorant that by the *vis nervosa* he everywhere implies that influence originating in the *brain* and spinal cord, which, being propagated along the nerves, causes muscular contraction, whether in obedience to volition, emotion, or any other stimulus. Haller's great object was to separate the idea of this *vis nervosa*, or nervous power, from the *vis insita,* or contractility of the muscle; and whilst, in the case of the heart and alimentary canal, he argues that the contraction is due to the direct excitement of the *vis insita,* he would say that, wherever a muscle is caused to contract by the influence of a nerve, it was by an operation of the *vis nervosa.* We repeat our assertion, then, that if the *vis nervosa* of Haller be intended by Dr. Hall, he has no right to limit it to his excito-motor system of nerves, since it is also the motor influence by which volition and emotion operate upon the muscles. If we are wrong, let Dr. Hall prove our error from Haller's works. His views on the *vis nervosa,* however, are undergoing so constant a change, that he would probably find it more convenient to abandon all reference to Haller, and give to the term a new definition. How, for instance, can the following be stated of a power which is defined as originating in the nervous centres, and as transmitted from them along the peripheral nerves? "I can discover no reason for thinking that the true spinal system differs, either in its essential structure or properties, in any of its parts, from its minute origins in the nervo-cutaneous and nervo-mucous surfaces, in its incident, in its central, in its reflex portions, or in its ultimate distributions in the muscular or nervo-muscular fibres. All is mere difference of *form,* mere morphology. The structure, the animating principle, is the same in each and every part, only modified in the incident, central, and reflected portions. *In every part of this system, the vis nervosa is seated, is operative.*" Again, "nerves are *not* mere *carriers.* A muscular nerve *has* the motor power within itself; so has the spinal marrow; and, as I have recently discovered and proved, so have the incident nerves." "We really know nothing of the matter," adds Dr. H., "except the *facts,* which *experi-*

*ment* and *observation* bring to light, however the biblio-physiologist may dogmatize on the subject." In this last assertion we most heartily concur; and we are quite willing to leave to our readers the decision of the following questions, by which it will be known who keeps closest to facts, and who dogmatizes least, ourselves or Dr. Hall.

1. Is it consistent with *anatomical* facts to assert that the *central* portion of the spinal system of nerves (and, of course, of the system correspondent to it in the invertebrata) differs from the nervous trunks only in *form?* or is it not rather the fact that we universally find the fibres of the incident nerves losing themselves, and those of the motor nerves originating, in a substance chiefly composed of a plexus of blood-vessels?

2. Is it not most consistent with physiological facts to assert that in this central nervo-vascular substance the motor influence originates, and that it may reasonably be regarded as of similar character, whether excited by a mental act of volition or emotion, or by a stimulus conveyed thither from the circumference; rather than to assert that volitions and emotions themselves travel along the motor nerves, and that the motor influence may originate independently of the central organs, and even travel *towards* them?*

In regard to the so-called *retrograde* course of the stimulus along the spinal cord, again, Dr. Hall greatly misrepresents our meaning, which was simply this: We fully admit that a stimulus conveyed to the spinal cord by an incident nerve, between its anterior and posterior extremities, or an irritation of the spinal cord itself in the same situation, may give rise to motion in either or both those parts; but we merely say that, looking at the true spinal cord in the light of a continuous chain of independent ganglia, we do not see that either of these actions is more direct or more retrograde than the other. Every one knows that the desire to evacuate the fæces or urine, caused by an impression conveyed to the lower part of the spinal cord by the afferent nerves, will cause a number of sympathetic or reflex contractions in the muscles of respiration, and in others which derive their motor nerves from the upper part of the column; and to speak of this as an instance of *retrograde* action, separating it from that to which it is most nearly allied, appears a pernicious use of terms, and calculated to lead into error. It *has* led Dr. Hall into what we believe the great error of supposing that a motor influence travels *towards* the centre, in the operation of the spinal system; at least, we can find no other ground for that opinion than facts analogous to this.

Further, we fully admit the importance of the fact in pathology; but we assert that it was well known long before Dr. M. Hall's researches on this subject commenced; as, for example, in the case of traumatic tetanus. (See vol. III, p. 39, and V, p. 539.)

Will Dr. Hall inform us which is the *direct* and which the *retrograde* course of the reflex action in the nervous circle of the radiata; or in the

* Dr. Hall may perhaps point to the well-known fact, that pinching or otherwise stimulating a motor nerve in its course will give rise to muscular contraction, and may thence argue that the *vis nervosa* is entirely independent of the central organ; but he will do well to remember that the same argument would hold in regard to the sensory nerves, and would go to prove that their extremities and the organs at which they terminate are not the real seat of the sensory impressions. For our own explanation of the fact, see vol. V. p. 494, note.

ganglionic column of the articulata? When the idea of a *head*, containing a brain or something analogous to it, is put out of the question (as, in studying the phenomena of the spinal system, we are called upon to do) we can see no reason why the operation of a stimulus applied to the centre of the spinal cord, or to one of the incident nerves of the same part, can be said to more *retrograde* when it sets the arms in motion, than when it affects the legs. And if this be not assumed, the doctrine that a motor impulse travels *towards* the spinal cord will be found, we believe, entirely destitute of support. But so far are we from wishing to dogmatize on the question, that we would prefer leaving the discussion of it to our readers themselves; and when Dr. Hall has adduced more satisfactory evidence on behalf of his view of the case, or can show that any two physiologists of note have embraced his views, we will give them once more our best consideration. And we trust that we have shown that we are not so wedded to error, even when it has been advocated by ourselves, as to be unwilling to receive truth. One excuse, however, we may subjoin, that it is not always easy to *understand* Dr. Hall's opinions. Let our readers judge for themselves from the following paragraph:

"To me it appears that the motor power acts along an incident nerve, as the vibrations of a ray of light or sound pursue their incident course; that the true spinal marrow induces a sort of polarization, and directs this motor force in new but defined sources of double or multiplied refraction or reflexion, inscrutable in their essence, and only to be ascertained by their effects in the destined and definite movements they induce." (Preface, p. xiv.)

No lover of physiological science can read Dr. Hall's present work, notwithstanding all its peculiarities, without feeling the mind introduced to the consideration of numberless questions of the most interesting nature. If the new facts presented are fewer than the author deems them to be, many old and familiar ones are placed in a new and striking light; and some parts of pathology, before vague and obscure, are illustrated with a clearness which is the more satisfactory because it is the evident result of legitimate and laborious research. Who can fail to lament that the just title thus raised by Dr. Hall to the gratitude of the profession and of posterity, should be impaired by countless manifestations of a fretfulness unworthy of the weakest mind; by an impatience of free criticism, carried even to a ridiculous excess; and by such frequent and such round assertions of his own merit as cannot but stamp him with the character of obtrusive and extravagant vanity as long as his book continues to be perused? Nothing less will satisfy Dr. Hall than to be placed at once side by side with Bacon, and Newton, and Herschel, and Haller, and Harvey. If any one presumes to doubt his possessing a mind of this exalted and capacious class, he rebukes the critic as ignorant and malignant; as youthful above all; forgetting that he himself aspired to be a teacher [of diagnosis] before he ceased to be a student, and that, in another part of the volume, he names, as among his reviewers in this Journal, one gentleman, at least, whom he must know to be more advanced in years and of higher professional standing than himself. There is scarcely a chapter of his work which is not disfigured by an eagerness to show that no former writer on the nervous system knew anything of the views to which his genius has attained; and that all sure nervous pathology must for evermore rest on the basis of his discoveries. Assuredly nothing can be less *Newtonian*, to use Dr. Hall's favorite expression, than these vaunting aspirations.

Engaged in a pursuit of undeniable dignity and importance, and adding useful matter to the stores of physiological and pathological knowledge, one might have hoped that the irritable system of Dr. Hall would have been calmed by the sweet influences of philosophy; but such is far from being the case. In every step of his great and useful investigation, he turns away his eyes from elevated subjects, casts them on earth's pavement, and hurls defiance and wrath and scorn at societies, and reviewers, and private rivals. Blind, apparently, to the high reward which awaits all diligent followers of truth, he bewails in unmanly terms, his exclusion from worldly rewards of honours or money, which, with characteristic candour, he avows that he thinks he has deserved. It is impossible for a man of science to exhibit himself in a more melancholy light than this. Yet such an exhibition of the workings of mere intellectual ambition may usefully show the utter worthlessness of mere intellectual endowment. There is a wisdom, we believe, as well as a happiness, which has its root in the affections and moral qualities; and where it flourishes, the mind expatiates freely and cheerfully beneath its modest shade. In our time this source of wisdom is too little sought. The fruit of knowledge is too often gathered as a mere means of procuring worldly distinction and advantage; and if these do not follow, the blessed fruit itself turns but to bitterness. Hence arise heart-burnings, and jealous controversies, and all that so often makes the minds of scientific men unamiable, and their lives a waste of sour dissatisfaction; and all that so often causes them, when collected into societies, to constitute but a community of angry and stinging insects, whose foibles and whose fierceness console and amuse those who possess no science at all. Even Dr. Hall, aspiring as he does to the fame of Bacon, forgets one at least of the limitations of knowledge set forth by that great and imperfect man in the outset of his work on its proficience and advancement: "that we make application of our knowledge to give ourselves repose and contentment, and not distaste or repining."

The man of science, to be happy, must pursue science with exalted aim; forget that there are Royal societies and books of periodical criticism; and, knowing how small a corner of the curtain that hides all truth from man's gaze can be lifted up by any one hand, should be charitable towards those labouring, like himself, for all time. In this spirit, with unfeigned sorrow we say it, Dr. Hall appears to us to be eminently wanting: and, great as his merits unquestionably are, and praiseworthy and admirable his labours, this defect will detract from his just reputation until, in the course of years, the trifles which trouble the surface of science are swept down the gulf-stream of human events, and the solid deposits alone remain to aid in the formation of the great continent of truth.

